# Analysis of Suspension of Clinical Trials for Drug Registration in China

**DOI:** 10.3389/fphar.2020.598574

**Published:** 2021-06-29

**Authors:** Xian Su, Xiaocong Pang, Xin Zeng, Yi Gao, Yimin Cui, Haixue Wang

**Affiliations:** ^1^ Department of Pharmacy, Peking University First Hospital, Beijing, China; ^2^ Office of Clinical Trial Management, Centre for Drug Evaluation, National Medical Products Administration, Beijing, China; ^3^ Office of Biostatistics and Clinical Pharmacology, Centre for Drug Evaluation, National Medical Products Administration, Beijing, China; ^4^ Department of Pharmaceutical Engineering, College of Pharmacy, Harbin University of Commerce, Harbin, China

**Keywords:** risk control system, pharmacovigilance, closed-loop treatment mechanism, safety, suspension

## Abstract

Suspension is an important risk control measure during clinical trials. We investigated the use of this in China and identified common reasons for suspension by analyzing trends, hold issues, outcomes, background and design characteristics of suspended clinical drug trials from January 1, 2013 to December 1, 2019. A total of 298 clinical trials during the study timeframe were registered, accounting for 3.1% of all clinical drug trials. Numbers and proportion of clinical trials suspended based on benefit/risk factors have been increasing without holds on registrations by Center for Drug Evaluation. Reasons for suspension vary among trial phases, benefit and risk factors, protocol issues etc. 67% of trials that have been on hold for >1 year were still on hold at the time of this analysis. Children and the elderly were enrolled in 4.1% and 41% of the suspended trials, respectively. Strengthening regulation of pre-market pharmacovigilance through optimizing reporting and monitoring of safety information during clinical trial is thus needed. Establishing a closed-loop treatment mechanism for trial suspension is also important. Examination of potential risks, such as the quality of protocols, the ability of the institution to support the trial, and the adequacy of supplies of the investigational product is needed before beginning clinical trials. More careful evaluation at the drug registration phase will reduce the frequency of suspension and protect subjects after suspension occurs.

## Introduction

The number of clinical trials in China has increased annually from 918 in 2014–2,756 in 2019, under the influence of policies to encourage innovation and drug evaluation reform (The General Office of the Central Committee of the Communist Party of China, 2017). Since July 27, 2018, China implemented an implied permission system for examination and approval of clinical trials to control risks more effectively while simplifying the approval process for clinical trials and improving subsequent evaluation (National Medical Products Administration, 2018a). More strict requirements are put forward for dynamic supervision of clinical trials to support suspension, restart and transfer to inactive status (China Food and Drug Administration, 2013; U.S. Food and Drug Administration, 2014; U.S. Food and Drug Administration, 2015; U.S. Food and Drug Administration, 2016; National Medical Products Administration, 2018a; Standing Committee of the national People’s Congress of the People’s Republic of China, 2019).

*The Drug Administration Law of the People's Republic of China* requires that when safety issues or other risks are found during a clinical trial, the sponsor will promptly adjust protocols, halt, or terminate the trial (National Medical Products Administration, 2018b). Numerous reasons for holds have emerged since January 1, 2013. In this study, we hope to put forward theoretical scientific and system design methods to monitor and reduce risks. We will provide a reference for sponsors to improve the quality of clinical trials, through quantitative and trend analyses of suspended clinical drug trials.

## Methods

We conducted a systematic retrospective analysis of suspension of clinical trials for drug applications between January 1, 2013, and December 1, 2019. To fulfill the scientific and moral obligations of researchers and regulatory agencies, the National Medical Products Administration (NMPA) issued a notice in 2013 that all drug clinical trials being done as registration trials must be registered on the NMPA Registration and Information Disclosure Platform for Drug Clinical Studies in China as of 2013, including phase 1–4 drug trials and bioequivalence studies. For trials initiated before 2013, but for which the related new drug application was unfinished, registration was required to be done retrospectively.

We included suspensions of all clinical drug trials, including trials put on hold for cause or passively, as well as trials never put on hold. Data processing used three steps. First, we used the “trial status” field for Chinese drug trials the NMPA archive database, which includes the following options: initiative hold, passive hold, and unavailable (NA). We then searched for clinical trials suspended since January 1, 2013. Finally, we downloaded the resulting XML dataset into a relational database (OracleRDBMS version 11.1 g Oracle Corporation) for summary analysis. If field information was blank, it was recorded as “missing.” Suspension of clinical trials in some countries and research centers were classified as “partial hold,” and others as “complete hold” National Medical Products Administration, 2018. We focused on analysis of four key indicators for suspension of clinical drug trials: trends, causes, outcomes, and selected characteristics. Frequency and percentage were assessed as category variables. Descriptive statistics will include counts and percentages for categorical data, and median, mean, SD, interquartile range, and range for continuous data.

### Trend Analysis of Clinical Trial Suspension

Trends in suspensions were analyzed by year of trial registration and year of hold. The ratio of suspensions to total trials was calculated as: trial suspensions by year/the total trials registered in the same year, the number of annual trial suspensions and the annual total trials are counted according to the year of registration of the trial; the ratio of annual cumulative suspensions was calculated as: cumulative suspensions/total number of trials, the number of cumulative suspensions and the total trials are counted between January 1, 2013, and the last day of the year.

### Analysis of Reasons for Suspension

Reasons for suspension were classified based on risk-benefit factors and based on non-risk-benefit factors, and the specific reasons were further subdivided (Table 1). We selected June 2016 as the median time point for overall analysis and for analysis of two data subsets based on timeframe (January 2013 to June 2016 and June 2016 to December 2019). Further, the reasons for holds at different stages of drug research and development (phase I, phase II, phase III) were analyzed.

**TABLE 1:** Classification of suspension justifications.

Discipline	Subcategory assigned to Reasons
Risk-benefit factors	Lack of therapeutic benefit
	Reach the endpoint prematurely
	Safety issues: adverse event, pharmaceutical risk signal, toxicological studies suggest significant safety risk signals
Non-risk-benefit factors	Protocol factors: protocol revision, deficiency of protocol design, protocol optimization
	Insufficient supply: shortage of experimental drugs or other supply chain problems
	Strategic factors: business factor, competitive environment, optimization and other factors
	Financial factors: lack of funds, delay or withdrawal are the main problems
	Research institution/researcher factors: infeasibility of research institution/researcheretc.
	Policy factors: changes in regulations, changes in registration requirements, and regulatory requirements
	Difficulty in recruiting subjects: low enrollment rate and difficulty in recruitment
	Quality risk factors: program violations and GCP suspension
	Pharmaceutical/preclinical factors: pharmaceutical/preclinical factors not related to safety
	Sponsors factors: reasons for hold determined by sponsors but not related to strategy
	BE inequivalence
	Others: the reason for hold is unclear and difficult to classify

### Analysis of Outcomes of Suspensions

Outcomes of suspension include premature termination, hold lifted, and remaining indefinite hold; trials not been expressly terminated or lifted were categorized as remaining on hold. Correlations between causes of suspension and suspension outcomes were assessed.

### Background and Design Characteristics of Suspended Clinical Trials

We performed analysis of background and design characteristics of suspension trial, including the drug type, indications, trial scope, funding sources, phase, Estimated Enrollment, DMC (Data Monitoring Committee) or not, children and the elderly entered or not.

The definition of children is "the age of the subjects is less than 18 years old", and the definition of the elderly is "the age of the subjects is more than 65 years old".

## Results

**FIGURE 1:**
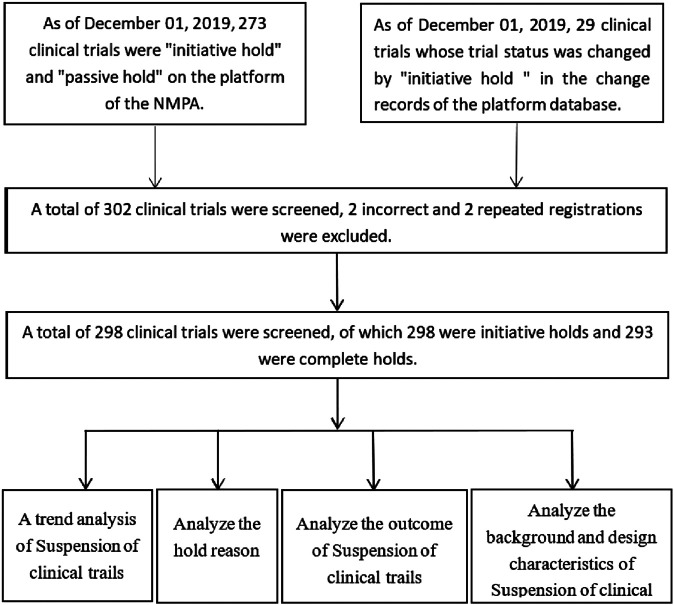
Data processing and key indicators.

A total of 298 registered clinical trials were suspended during the study timeframe, accounting for 3.1% of all clinical drug trials (N = 9,542), and all 100% were suspended purposefully by sponsor. Four partial holds were placed because the international multicenter has completed enrollment but the Chinese research did have not enroll subjects; one partial hold was placed in response to subject input. The remaining 293 clinical trials were complete holds (Figure 1).

### A Trend Analysis of Suspension of Clinical Trials

**FIGURE 2:**
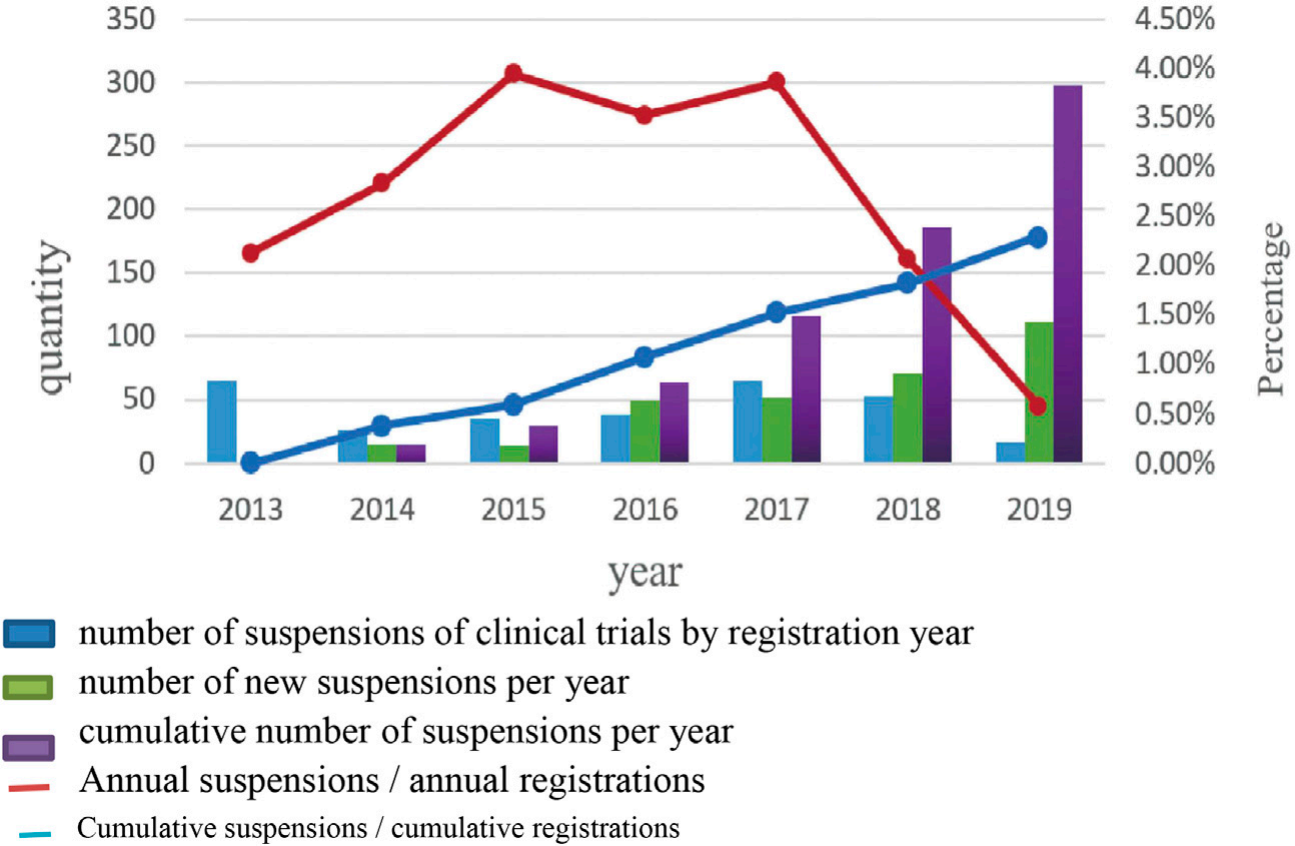
A trend analysis of suspensions of clinical trials.

No obvious trends in the number of suspensions by year of registration or ratio of suspensions to total trials were evident. These ratios varied between 0.58% and 3.95%. The annual numbers and cumulative numbers of suspensions by hold year increased annually, and the proportion of cumulative annual increased from 0% to 2.3% (Figure 2).

### Analysis of Reasons for Suspensions

Between January 1, 2013, and December 1, 2019, the proportion of suspensions of risk-benefit factors was 22.1%, of which safety issues accounted for 7.5%, and lack of therapeutic benefit 13.1% (Table 2). The proportion of suspensions based on non-risk-benefit factors was 79.79%, including strategic issues (16.7%), protocol difficulties (14.2%), BE inequivalence (9.6%), difficulty in recruiting subjects (10.3%), researcher/research institution limitation (5.7%), policy factors (6.7%), supply problems (4.3%), financial issues (2.5%), and pharmacy/preclinical factors (1.4%), with other factors making smaller contributions.

**TABLE 2:** Analysis of reasons for suspensions overall and clinical trial phases.

No./Total no. (%)						
		All suspension of clinical trailss	Phase I^a^	Phase II^a^	Phase III^a^	BE
		(N = 298)	(n = 49)	(n = 57)	(n = 82)	(n = 91)
Risk-benefit factors^b^		57/258 (22.1)				
	Safety issues	21/282 (7.5)	9/47 (19.15)	3/56 (5.36)	6/76 (7.89)	2/85 (2.35)
	Lack of therapeutic benefit	37/282 (13.2)	6/47 (12.77)	5/56 (8.93)	24/76 (31.58)	2/85 (2.35)
Non-risk-benefit factors^b^		225/282 (79.79)				
	Protocol factors	40/282 (14.18)	6/47 (12.77)	11/56 (19.64)	8/76 (10.53)	15/85 (17.65)
	Supply factors	12/282 (4.26)	0/47 (0)	2/56 (3.57)	2/76 (2.63)	4/85 (4.71)
	Strategic factors	47/282 (16.67)	10/47 (21.28)	6/56 (10.71)	14/76 (18.42)	18/85 (21.18)
	Financial factors	7/282 (2.48)	1/47 (2.13)	6/56 (10.71)	0/76 (0)	0/85 (0)
	Research institution/researcher factors	16/282 (5.67)	6/47 (12.77)	2/56 (3.57)	1/76 (1.32)	7/85 (8.24)
	Policy factors	19/282 (6.74)	1/47 (2.13)	5/56 (8.93)	3/76 (3.95)	5/85 (5.88)
	Difficulty in recruiting subjects	29/282 (10.28)	6/47 (12.77)	8/56 (14.29)	7/76 (9.21)	3/85 (3.53)
	Quality risk factors	6/282 (2.13)	0/47 (0)	2/56 (3.57)	1/76 (1.32)	2/85 (2.35)
	Pharmaceutical/preclinical factors	4/282 (1.42)	1/47 (2.13)	0/56 (0)	0/76 (0)	3/85 (3.53)
	Sponsors factors	12/282 (4.26)	1/47 (2.13)	4/56 (7.14)	4/76 (5.26)	2/85 (2.35)
	BE inequivalence	27/282 (9.57)	0/47 (0)	0/56 (0)	0/76 (0)	27/85 (31.76)
	Others	9/282 (3.19)	1/47 (2.13)	4/56 (7.14)	4/76 (5.26)	4/85 (4.71)
Missing		16/298 (5.37)	2/49 (4.08)	1/57 (1.75)	8/82 (9.7)	1/91 (1.1)

^a^ I/II,II/III phase trials were excluded.

^b^Since categories are not mutually exclusive, the sum of percentages may not be equal to 100%.

Proportions of suspensions of clinical trials in phases I, II, III and BE due to risk-benefit factors was 32.6%, 14.5% 41.4%, and 4.9%, respectively. The most common reason in phase I studies were protocol differences (21.3%), safety issues (19.2%), and researcher/research institution limitations (12.8%). Suspensions in phase II were primarily due to protocol factors (19.6%), difficulties in recruiting subjects (14.3%), financial difficulties (10.7%), and lack of therapeutic benefit (7.9%). Lack of therapeutic benefit (34.3%) caused the largest proportion suspension phase III studies. Other notable reasons included strategic factors (18.4%), protocol problems (10.5%), difficulties in recruiting subjects (9.2%), and safety issues (7.9%). The main reasons for suspensions of clinical trials in BE were BE inequivalence (31.76%), strategy factors (21.2%), protocol difficulties (17.7%), and research institution limitations (8.2%).

During two periods from January 1, 2013 to March 4, 2016, and from March 4, 2016 to November 21, 2019, the proportion of total suspensions due to risk-benefit factors and safety issues increased in total number of registrations trials (Table 3). The proportion of suspensions based on risk-benefit factors decreased in otal number of suspension of clinical trials, but the percentage due to safety issues increased from 0 to 8.4%.

**TABLE 3:** Trend analysis of the timeline of suspensions of clinical trials.

No./total no. (%)					
		2013.01.01–2016.06.01	2016.06.01–2019.12.01	2013.01.01–2016.06.01	2016.06.01–2019.12.01
		Cumulative total number of registrations (n = 4,499)	Cumulative total number of registrations (n = 9,542)	Total number of suspension of clinical trailss (n = 31)	Total number of suspension of clinical trailss (n = 267)
Risk-benefit factors^a^		11/4,499 (0.21)	56/9,542 (0.59)	11/31 (35.48)	45/251 (17.93)
	Safety issues	0/4,499(0)	21/9,542 (0.22)	0/31(0)	21/251 (8.37)
	Lack of therapeutic benefit	11/4,499 (0.21)	37/9,542 (0.39)	11/31 (35.48)	26/251 (10.36)
Non-risk-benefit factors^a^		20/4,499 (0.45)	267/9,542 (2.80)	20/31 (64.52)	247/251 (98.41)
	Protocol factors	1/4,499 (0.02)	35/9,542 (0.37)	1/31 (3.23)	34/251 (13.55)
	Supply factors	0/4,499(0)	11/9,542 (0.12)	0/31(0)	11/251 (4.38)
	Strategic factors	7/4,499 (0.16)	52/9,542 (0.54)	7/31 (22.58)	45/251 (17.93)
	Financial factors	2/4,499 (0.04)	7/9,542 (0.07)	2/31 (6.45)	5/251 (1.99)
	Research institution/researcher factors	2/4,499 (0.04)	16/9,542 (0.17)	2/31 (6.45)	14/251 (5.58)
	Policy factors	2/4,499 (0.04)	13/9,542 (0.14)	2/31 (6.45)	11/251 (4.38)
	Difficulty in recruiting subjects	3/4,499 (0.06)	24/9,542 (0.25)	3/31 (9.68)	21/251 (8.37)
	Quality risk factors	1/4,499 (0.02)	6/9,542 (0.06)	1/31 (3.23)	5/251 (1.99)
	Pharmaceutical/preclinical factors	0/4,499(0)	4/9,542 (0.04)	0/31(0)	4/251 (1.59)
	Sponsors factors	2/4,499 (0.04)	12/9,542 (0.13)	2/31 (6.45)	10/251 (3.98)
	BE inequivalence	0/4,499(0)	26/9,542 (0.23)	0/31(0)	26/251 (10.36)
	Others	2/4,499 (0.04)	9/9,542 (0.09)	2/31 (6.45)	7/251 (2.79)
Missing		0/4,499(0)	16/9,542 (0.17)	0/31(0)	16/267 (6.37)

^a^ Since categories are not mutually exclusive, the sum of percentages may not be equal to 100%.

During these same timeframes, the proportion of suspended trials based on non-risk-benefit factors increased overall. The proportion due to protocol factors increased from 3.2% to 13.6%, the proportion due to supply factors from 0% to 4.4%, and the proportion due to BE inequivalence from 0% to 10.7%. In contrast, proportion due to lack of therapeutic benefit decreased from 25.8% to 10.4%, and the proportion due to financial factors decreased from 6.5% to 2.0%.

### Analysis of Outcomes of Suspension

Among suspended trials, with four exceptions in China, 80 (27.2%) were terminated prematurely, 192 (64.0%) were still on hold, 26 (8.8%) had suspensions lifted, and 147 (49.3%) remained on hold for more than one year (Table 4). In this latter group, only 2.0% of suspensions had been lifted. Another 27.9% were terminated prematurely, and 67.3% remained on hold. The proportions trials still on hold were caused by safety issues, and lack of therapeutic benefit, 76.2% and 62.2%, respectively, and one trial in each group had been restarted clinical (Table 3).

**TABLE 4:** Analysis of outcomes of suspensions of clinical trials.

	All studies^a^ (N = 294)	Hold duration>1 year (n = 147)	Safety issues (n = 21)	Lack of therapeutic benefit (n = 37)
On hold	188/294 (63.94)	99/147 (67.35)	16/21 (76.19)	23/37 (62.16)
Terminate prematurely	80/294 (27.21)	41/147 (27.89)	4/21 (19.05)	13/37 (35.14)
Being restarted	26/294 (8.84)	3/147 (2.04)	1/21 (4.76)	1/37 (2.70)

^a^ Four multinational multicenter trials suspended only in China due to competition were excluded.

### Background and Design Characteristics of Suspended Trials

**FIGURE 3:**
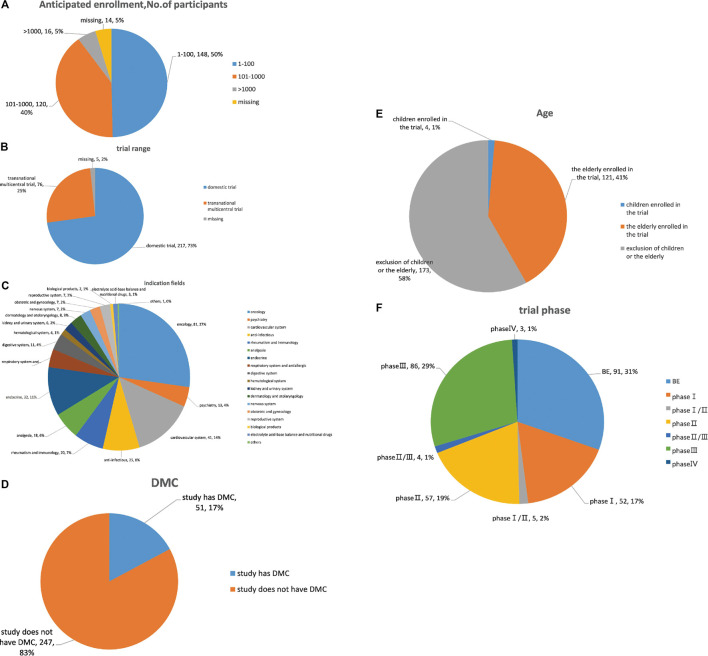
Analysis of clinical trial characteristics of suspended trials. **(A)** Drug type analysis of suspended trials; **(B)** indication fields analysis of suspended trials; **(C)** range analysis of suspended trials; **(D)** phase analysis of suspended trials; **(E)** age analysis of subjects in suspended trials; **(F)** DMC analysis of suspended trials; **(G)** sample analysis of suspended trials.

Overall, chemical drugs trials (n = 216, 73.7%) accounted for the majority of all suspended trial. By subcategory, proportions were: oncology (27.2%), cardiovascular (13.8%) and endocrine trials (10.7%) (Figure 3A). Domestic trials accounted for 70.1%, and transnational multicentral trials accounted for 25.9% of all trials. Further, BE accounted for the largest proportion of suspensions (30.5%), with percentages in other phases of 28.9% in phase III, 19.1% in phase II, and 17.5% in phase I. A large majority of suspended trials were utterly self-financed (94.5%); 17.1% had DMC; 42.3% and 5.6% planned enrollment of 101–1,000 subjects and more than 1,000 subjects, respectively; 4.1% and 41% specifically recruited children and the elderly, respectively.

## Discussion

This study is first to analyze the suspension of clinical trials for drug applications in China, to the best of our knowledge. Establishment of a reasonable mechanism for suspension will improve dynamic supervision of clinical trials while implementing the implied licensing system (National Medical Products Administration, 2018a; National Medical Products Administration, 2018b). A reasonable suspension mechanism requires not only standards for risk-based and non-risk-based suspensions, but also a closed-loop process from pre-suspension communication to suspension, resumption, or early termination (Boudes, 2015; Jarow et al., 2016; State Administration for Market Regulation, 2020). Thus, analysis of trends, causes, and outcomes of suspension of clinical trials is needed to support such a system.

Our results show no increasing trend in the number of suspensions of clinical trials by year of registration, but annual numbers and the cumulative proportion of suspended clinical trials increased year-by-year. We demonstrate that these changes may be related to significantly increased number of clinical trials and more standardized regulatory measures for clinical trials china (Li et al., 2019). At present, all suspensions of clinical trials in the NMPA Registration and Information Disclosure Platform for Drug Clinical Studies were put on hold by trial sponsors. We found that 20.9% of trials were put on hold because of risk-benefit factors, and the proportion of safety issues increased, of which 90.5% were due to adverse events. Implementation of E2A, M1, and E2B in China will promote reporting of drug surveillance information and position China’s adverse drug reaction monitoring in line with international standards (Jarow et al., 2017). Achieving this goal will allow China to share drug safety data with the United States, Japan, and other ICH members (Califf et al., 2012; Jarow et al., 2017; Li et al., 2019; Manning et al., 2019).

The NMPA issued the Notice of the General Administration on the Application of the second-level guiding principles of the International Technical Coordination Committee for the Registration of Human drugs and decided to apply the five second-level guiding principles of the International Technical Coordination Committee for Human Drug Registration, E2A, E2D, M1, and E2B (European Commission, 2010). The notice establishes the basis for effective, dynamic evaluation and monitoring of safety issues in clinical trials. Therefore, sponsors need to improve their drug surveillance systems and submit timely safety reports.

Our findings show that 76.19% of clinical trials that were suspended for safety issues are still on hold, and all were put on hold by sponsor. When the European Union suspends or prematurely terminates a trial due to a change in risk-benefit balance, restarting the trial is required after substantive revision (Get et al., 2015). At present, no relevant regulations exist for lifting suspensions of clinical trials in China. . Attention to lifting of suspensions of clinical trials is recommended.

Strategic factors have been the most common cause for suspension since 2013 in China, and are also the most prevalent reason for halting phase I trials. The publicly accessible information on the website of NMPA Registration and Information Disclosure Platform for Drug Clinical Studies includes sponsor information, trial design, indications, researcher information, and trial status since 2013. Finally, sponsors will gain insight for saving resources for drugevaluation and improving the efficiency of drug research and development. We found that the main reasons for the suspension of clinical trials based on non-risk-benefit factors, included elements that affect the quality of trials, such as unreasonable protocol design, lack of researcher/research institutions capability, and insufficient supply of experimental drugs or other substances. Protocol factors, the foundation of high-quality clinical trials from both science and feasibility viewpoints, was one a primary factor for suspensions in all phases of clinical trials. Tufts Center for the Study of Drug Development (TuftsCSDD) in the United States concluded that most program revisions could be avoided at the design stageby analyzed 984 associated program revisions (Jarow et al., 2017). In this study, difficulty in recruiting subjects was also a primary factor for suspensions in each phase. Commonly, this difficulty was that entry standards were too strict. Sponsors should improve clinical designs to avoid deficiencies that affect the feasibility of completing the trial.

Similarly, researchers/research institution limitations, supply issues, and other quality factors are also noteworthy. Often problems with researchers/research institutions were due to lack of feasibility rather than the dereliction of duty. *Record system of Clinical Drug Trial Institutions* issued in China will effectively alleviate the shortage of clinical research resources. Finally, insufficient supplies of experimental drugs accounted for 82.3% of all supply factors; sponsors must ensure an adequate supply of experimental drugs. Lack of drug(s) affects the reliability of clinical trial data and the rights and interests of subjects.

Only a minority of trials subject to suspension will be able to resume, and 33.2% of suspended clinical trials have been on hold for more than one year. We believe that it is necessary to consider whether closed-loop procedures for suspended clinical trials with hold durations limited to a certain period, such as drawing lessons from the FDA's practice of holds ordered passively. Suspensions, in this practice, that last for more than one year are automatically transferred to a static state (Lapteva and Pariser, 2012). If the static state persists for more than one year, the trial is automatically terminated prematurely.

The drug categories for suspensions were consistent with statistics of the clinical drug trial registration platforms; proportions of suspensions in international multicenter and phase III clinical trials was higher than that of all trials, indicating a relationship between trial complexity and suspension. Our results showed that 42.3% of suspended trials planned to include up to 1,000 subjects and 5.6% planned to include more than 1,000 subjects, and the elderly were targeted in 40.6% of the trials. Sponsors need to consider suspension of clinical trials from an ethical point of view and for protection of study participants.

## Conclusion

This study demonstrates that the number of suspensions of clinical trials and percentage of suspensions based on safety issues were increasing year-by-year, and a large proportion of clinical trials remain on hold. Protocols, researcher/research institution, the supply of drugs, and other factors were the main causes of suspension.

The results of this study are important for improving the ability of dynamic management of clinical trials for drug registrations and providing suggestions for the system design of evaluating suspension of clinical trials. Also, results provide a reference for preventing and reducing safety and quality risks in clinical trials, and especially highlight the importance of improving protocol design.

Some limitations in this study are noted. First, only holds of clinical trials were address, and not common pharmaceutical and non-preclinical study defects that lead to suspensions of IND applications and trials. In this regard, the present study is not a comprehensive reference for IND applications. Second, lack of full information for registered clinical trials prevented us from conducting a more in-depth discussion on the characteristics of clinical trials, which might be considered in future studies.

## Data Availability

The raw data supporting the conclusions of this article will be made available by the authors, without undue reservation
